# Original method in the treatment of varicose veins of the lower limbs in elderly patients candidates for orthopedic surgery

**DOI:** 10.1186/1471-2318-11-S1-A21

**Published:** 2011-08-24

**Authors:** L Goffredi, PF Atelli, M Apperti, MD Della Rocca, S Apperti

**Affiliations:** 1 Pathophysiology and Therapy of venous diseases, Department of Anesthetic, Surgical and Emergency Sciences, Inter-University Centre for research and training in Phlebology, II University of Naples, Italy; 2 Collaboration in the development of the equipment Visioven®

## Background

The increase in lower limb orthopedic surgery in the elderly patients often leaves the vascular surgeon faced with a growing demand for treatment of varicose veins of the lower limbs, in order to reduce the risk of post-operative thromboembolic. The laser treatment allows us to reach the goal with the advantage of reduced invasiveness and reduced recovery times, greatly facilitating subsequent orthopedic therapy.

## Materials and methods

We have used this method of treatment since 2006 on about 40 geriatric patients with varicose veins and lower limb orthopedic surgery candidates. A laser with probe 600 µs m. and a light source tested and patented by us (Visioven ®) was used on all patients. The development of this equipment was necessary so that the operation can not happen "blindly" but was targeted. The instrument consists of a laser light source powered by a "control box" that contains the drivers for this laser source. The handpiece, autoclavable, is provided on the breech with the laser diode block, while on the tip of a defocusing lens that achieves the maximum luminous intensity of contact and appropriate dispersion already at a distance of 10 cm.

## Results

No patient had post-operative complications and during the follow-up no patient showed signs of recanalization of the treated vases. The period of time between the intervention of laser photocoagulation and the following orthopedic intervention was in average of 15 days.

## Conclusions

The treatment of varicose veins of the legs with EVLT (EndoVenous Laser Therapy) is by now a methodic of a great success.

In this context, the Visioven ® has proven extremely useful, since the laser light can cross better than other light sources, to ensure the treatment of varicose veins under direct vision. Our method allows the insertion of the probe, either directly through a thin needle, thus avoiding having to perform a number of skin incisions and removing the risk of thermal injury due to exposure to incongruous laser energy outside the venous lumen.

In conclusion, the laser photocoagulation treatment under direct vision through Visioven® is in our view an effective method, targeted and rapid treatment of varicose veins of the lower limbs in geriatric patients who are candidates for orthopedic surgery [1,2].
